# Primary Skeletal Muscle Lymphoma Presenting as Acute Compartment Syndrome of the Forearm: A Case Report

**DOI:** 10.7759/cureus.93706

**Published:** 2025-10-02

**Authors:** Kazuhiro Ikeda, Hiromitsu Tsuge, Sho Kohyama, Naoya Kikuchi, Takaji Yanai

**Affiliations:** 1 Department of Orthopedic Surgery, Institute of Medicine, University of Tsukuba, Tsukuba, JPN; 2 Department of Orthopedic Surgery, Kikkoman General Hospital, Noda, JPN

**Keywords:** compartment syndrome, diffuse large b-cell lymphoma, dlbcl, fasciotomy, forearm, lymphoma

## Abstract

Primary skeletal muscle lymphoma rarely causes compartment syndrome. We encountered a case of diffuse large B-cell lymphoma that presented as acute compartment syndrome. A 70-year-old woman developed subacute swelling and pain in the left forearm without an identifiable cause. Compartment pressures were markedly elevated at 110 mmHg in the dorsal and 125 mmHg in the deep volar compartments. Despite emergent fasciotomy, her symptoms continued to progress. Intraoperative muscle biopsy revealed diffuse large B-cell lymphoma with overexpression of *MYC *and *BCL2. *After the initiation of R-CHOP (Rituximab, Cyclophosphamide, Doxorubicin, Vincristine, and Prednisone) chemotherapy, the forearm swelling and pain subsided, and the fasciotomy wound was managed conservatively until closure. Because of the high-risk double-expressor profile, the regimen was escalated to EPOCH-R (Etoposide, Prednisone, Oncovin (Vincristine), Cyclophosphamide, hydroxydaunorubicin (Doxorubicin), and Rituximab), and six courses achieved complete remission. The patient experienced no recurrence during four years of follow-up and developed no sequelae related to compartment syndrome. This case highlights the importance of rapid diagnosis through tissue sampling at the time of fasciotomy. Prompt initiation of chemotherapy is directly linked to both survival and preservation of limb function.

## Introduction

Skeletal muscle involvement by malignant lymphoma is rare, accounting for approximately 0.1-1.4% of all extranodal lymphomas [1]. In most cases, it reflects systemic multi-organ dissemination. Primary skeletal muscle lymphoma is even rarer, representing only about 0.1% of all lymphomas [2]. Despite its rarity, it is a clinically important condition that may first present to orthopedic surgeons. Although early initiation of chemotherapy is crucial for survival, diagnosis is often delayed and the overall prognosis remains poor [3-6].

A major reason for this delay is the nonspecific clinical presentation. Patients typically present with swelling and pain, but these manifestations closely resemble those of infection, inflammatory myopathies, or vascular disorders [3-5,7,8]. Consequently, it is often misinterpreted, and opportunities for timely therapeutic initiation may be missed.

We report a case of diffuse large B-cell lymphoma infiltrating skeletal muscle that presented as acute compartment syndrome of the forearm. In this patient, acute compartment syndrome was appropriately managed with prompt fasciotomy. However, symptoms continued to worsen even after decompression, ultimately necessitating systemic chemotherapy for the underlying lymphoma. This case highlights the diagnostic challenges of primary skeletal muscle lymphoma and emphasizes the need to consider malignant disease in the differential diagnosis of unexplained compartment syndrome.

## Case presentation

A 70-year-old woman presented with left forearm pain to the outpatient clinic. She had a history of rheumatoid arthritis and was taking prednisolone 5 mg/day, methotrexate 8 mg/week, and sulfasalazine 1,000 mg/day. Two months before the presentation, she received a COVID-19 vaccine. We noted diffuse swelling of the left forearm without warmth, erythema, or discrete point tenderness. She had a full, painless, active range of motion of the wrist and fingers. The condition was judged to be non-urgent, and magnetic resonance imaging (MRI) and clinical follow-up were scheduled.

Two weeks later, atraumatic swelling and pain developed in the left forearm and progressively worsened, leading her to present again to our hospital. The patient complained of unbearable pain and worsening of her symptoms. Her entire forearm was swollen with diffuse induration. However, she fulfilled only one of the five classic signs of compartment syndrome [9,10]: the presence of severe pain, while the radial artery was palpable, finger skin color was preserved, sensation was intact, and active finger motion remained. Compartment pressures were 6 mmHg (radial), 110 mmHg (dorsal), 34 mmHg (superficial volar), and 125 mmHg (deep volar), while the blood pressure was 128/88 mmHg. These values indicated marked elevation in the dorsal and deep volar compartments, with delta pressures (ΔP = diastolic pressure − intracompartmental pressure [10]) of −22 mmHg and −37 mmHg, respectively. 

**Figure 1:**
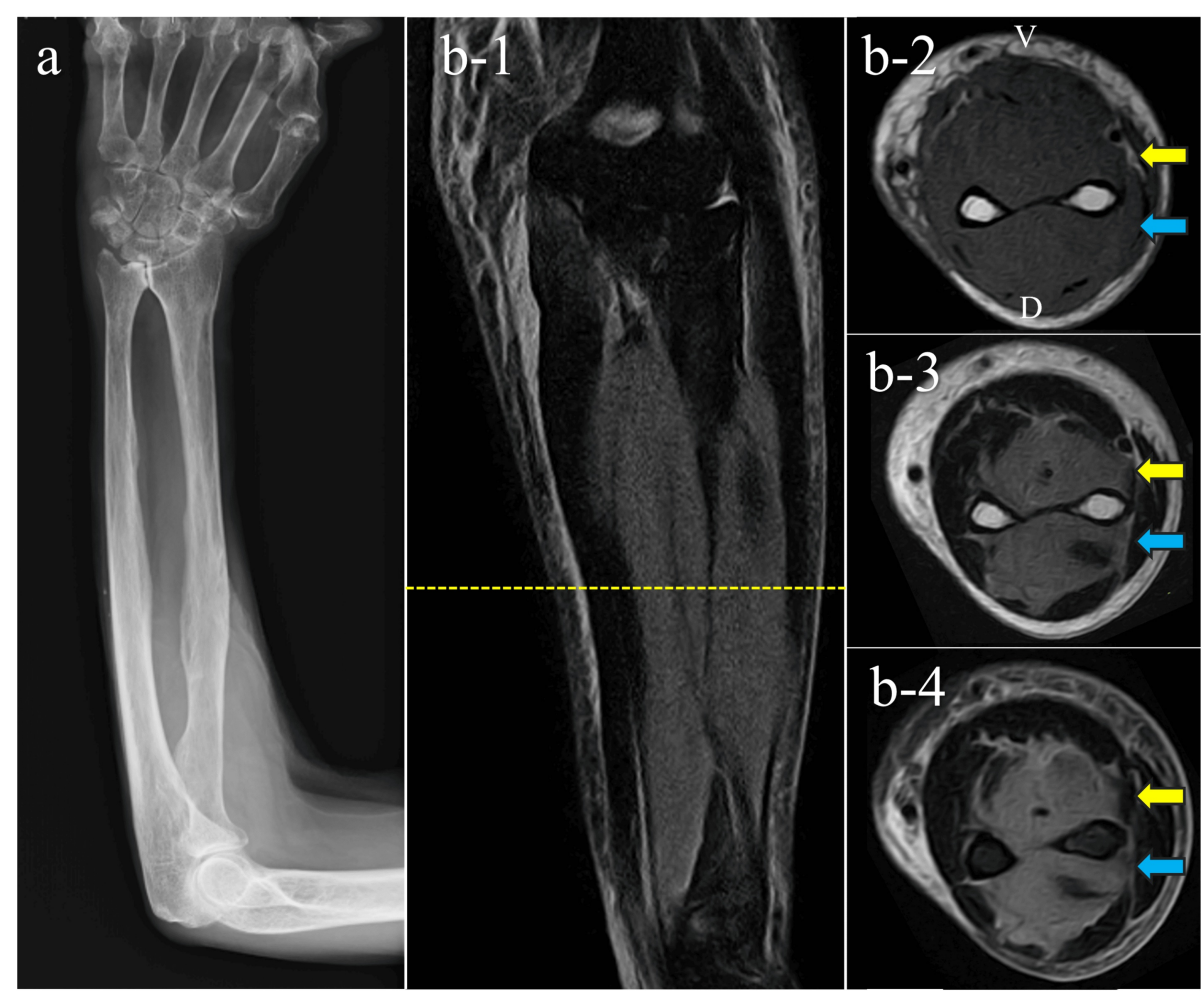
Imaging studies done at the second visit. (a) Plain radiograph of the forearm; (b) MRI of the forearm: (b-1) STIR coronal image, (b-2) T1-weighted axial image, (b-3) T2-weighted axial image, (b-4) STIR axial image. V, volar side; D, dorsal side; MRI, magnetic resonance imaging; STIR, short tau inversion recovery. Yellow dotted line indicates the slice level corresponding to images b-2 to b-4. Yellow arrows indicate the lesion in the deep volar compartment. Blue arrows indicate the lesion in the dorsal compartment.

MRI was done, which revealed diffuse signal alteration in the dorsal and deep volar compartment muscles, showing isointensity on T1-weighted images and hyperintensity on both T2-weighted and short tau inversion recovery (STIR) sequences (Figure 1). Blood tests showed no hematologic abnormalities, no marked inflammatory elevation, and a normal creatine kinase level (Table 1).

**Table 1:** Laboratory findings WBC, white blood cell count; PT, prothrombin time; INR, international normalized ratio; APTT, activated partial thromboplastin time

Parameters	Patient Value	Reference Range	Unit
WBC	8000	4500–8000	/µl
Segmented neutrophil	90	40–74	%
Lymphocyte	7.1	18–59	%
Monocyte	2.1	0–8	%
Eosinophil	0.4	0–6	%
Basophil	0.4	0–2	%
Hemoglobin	13	12–16	g/dl
Platelet count	42.1	14–34	10⁴ /μl
Aspartate aminotransferase	41	7–38	U/L
Alanine aminotransferase	38	4–43	K/L
Alkaline phosphatase	83	103–335	U/L
Blood urea nitrogen	12.7	8–20	mg/dl
Creatinine	0.61	0.36–1.06	mg/dl
Creatine phosphokinase	54	50–200	U/L
C-reactive protein	1.3	0–0.3	mg/dl
Procalcitonin	0.03	0–0.05	ng/ml
PT	12.3	10.5–13.5	sec
PT-INR	1.04		
APTT	29.6	26.1–35.6	sec

Surgery

**Figure 2:**
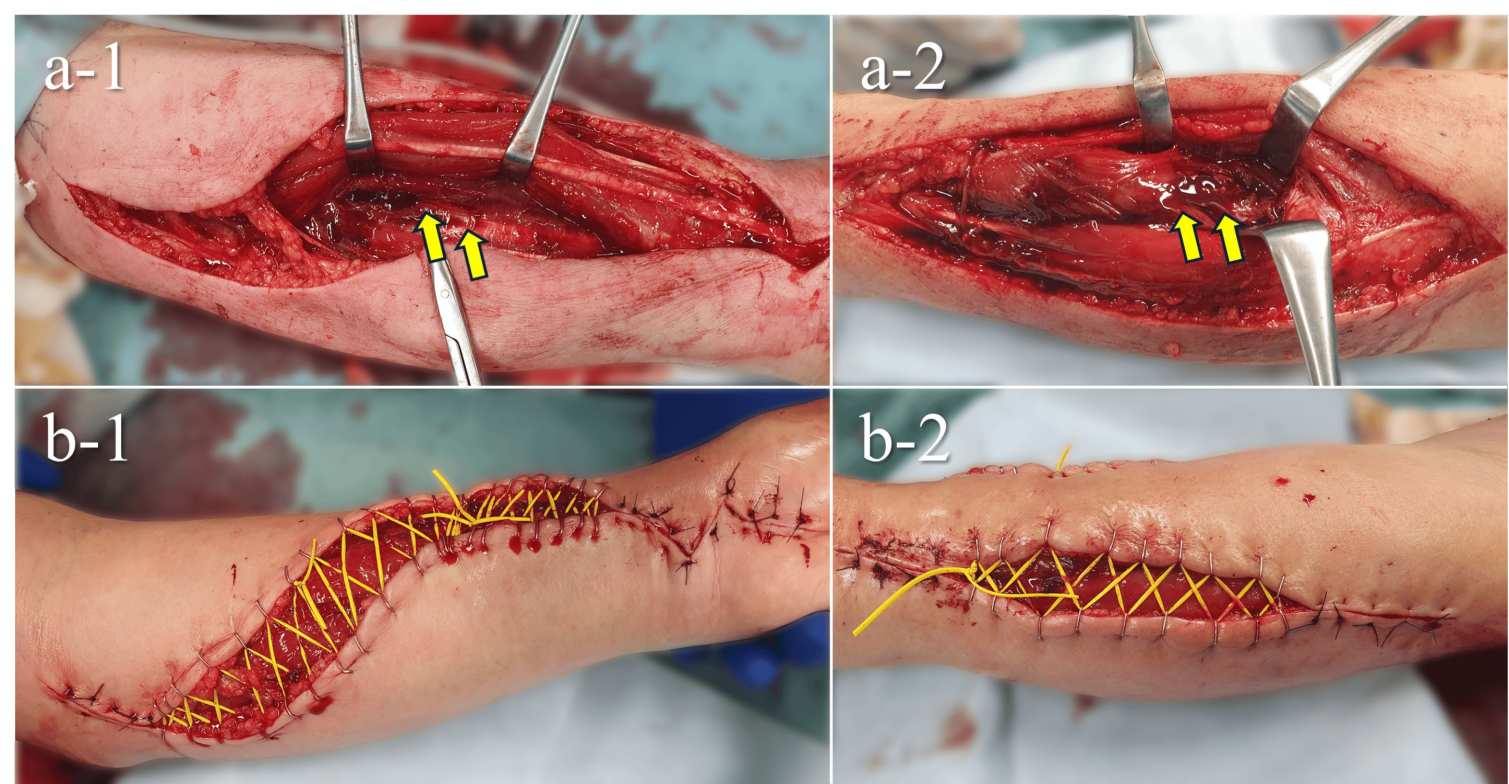
Intraoperative findings at fasciotomy. (a-1) Volar compartment release showing hematoma within the FDP and FPL (yellow arrows), (a-2) Dorsal compartment release showing hematoma within the EDC (yellow arrows), (b-1) Shoelace suture on the volar side, (b-2) Shoelace suture on the dorsal side. FDP, flexor digitorum profundus; FPL, flexor pollicis longus; EDC, extensor digitorum communis

Fasciotomy was performed emergently for the progressive compartment syndrome. Under regional anesthesia, we released all compartments of the left forearm and the carpal tunnel (Figure 2). The dorsal and deep volar muscles appeared edematous, consistent with the preoperative findings. The flexor digitorum profundus (FDP), flexor pollicis longus (FPL), and extensor digitorum communis (EDC) showed grayish discoloration, suggesting myoglobin depletion as well as intramuscular hematoma. There were no obvious signs of infection, such as pus or necrotic granulation tissue, within the muscle. We submitted muscle samples from the FDP and EDC for pathological examination. The wound was managed with shoelace sutures and negative-pressure wound therapy (NPWT).

Postoperative course

**Figure 3:**
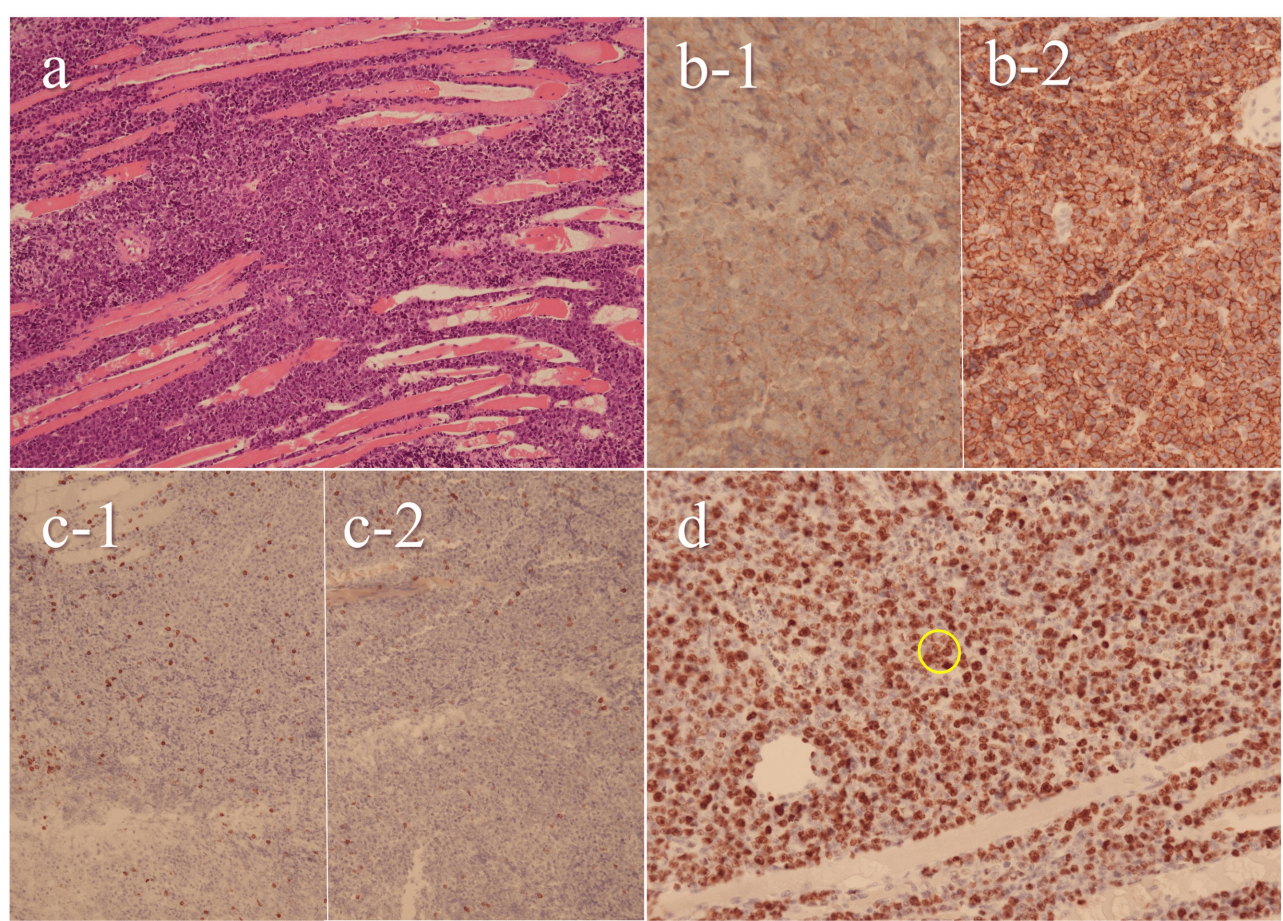
Histopathological findings. (a) Hematoxylin and eosin staining showing diffuse proliferation of lymphoid cells between skeletal muscle fibers; (b) Immunohistochemistry for B-cell markers (b-1: CD10; b-2: CD20): diffuse membranous positivity in neoplastic lymphoid cells (brown, DAB); (c) Immunohistochemistry for T-cell markers (c-1: CD3, c-2: CD5): negative; (d) Ki-67 immunohistochemistry shows nuclear labeling in approximately 70% of tumor cells (Ki-67 index: 70%). Yellow circle indicates representative positive nuclei. These findings led to the diagnosis of diffuse large B-cell lymphoma.

On postoperative day (POD) 5, despite continued NPWT and icing after surgery, swelling and pain progressively worsened, with the forearm circumference reaching 29.5 cm. Therefore, we discontinued NPWT and removed the shoelace sutures. On POD 9, the patient developed numbness in the median nerve distribution, and the forearm circumference increased to 32.5 cm. Blood tests showed a mild elevation of soluble interleukin-2 receptor (sIL-2R, 1,360 U/mL). Histopathological examination of the intraoperative muscle biopsy revealed infiltration of atypical B lymphocytes, leading to the diagnosis of DLBCL (Figure 3).

On POD 12, she was transferred to a medical center with a hematology department for treatment of DLBCL. Because of progressive swelling, the volar fasciotomy was extended proximally, and an additional intraoperative specimen was obtained. Although Epstein-Barr encoding region in situ hybridization (EBER-ISH) was negative, methotrexate-associated lymphoproliferative disorder (MTX-LPD) could not be ruled out. Therefore, methotrexate was discontinued.

On POD 14, prednisolone (60 mg/day) was started, resulting in a slight improvement of forearm swelling and pain. On POD 24-31, R-CHOP (Rituximab, Cyclophosphamide, Doxorubicin, Vincristine, and Prednisone) chemotherapy was started at 70% of the standard dose. Prednisolone was tapered to the maintenance dose of 5 mg/day for rheumatoid arthritis. After the initiation of the R-CHOP therapy, swelling of the forearm gradually subsided. The fasciotomy wound was managed with wet dressings by the plastic surgery team and eventually closed spontaneously. The numbness in the median nerve distribution also resolved.

On POD 36, chromosomal analysis of the lymph node demonstrated expression of both the *MYC* and *BCL2* genes, leading to the DLBCL with a double-expressor phenotype. To address the double-expressor phenotype of DLBCL, the regimen was changed to EPOCH-R (Etoposide, Prednisone, Oncovin (Vincristine), Cyclophosphamide, hydroxydaunorubicin (Doxorubicin), and Rituximab), and a total of six courses were administered over three months.

**Figure 4:**
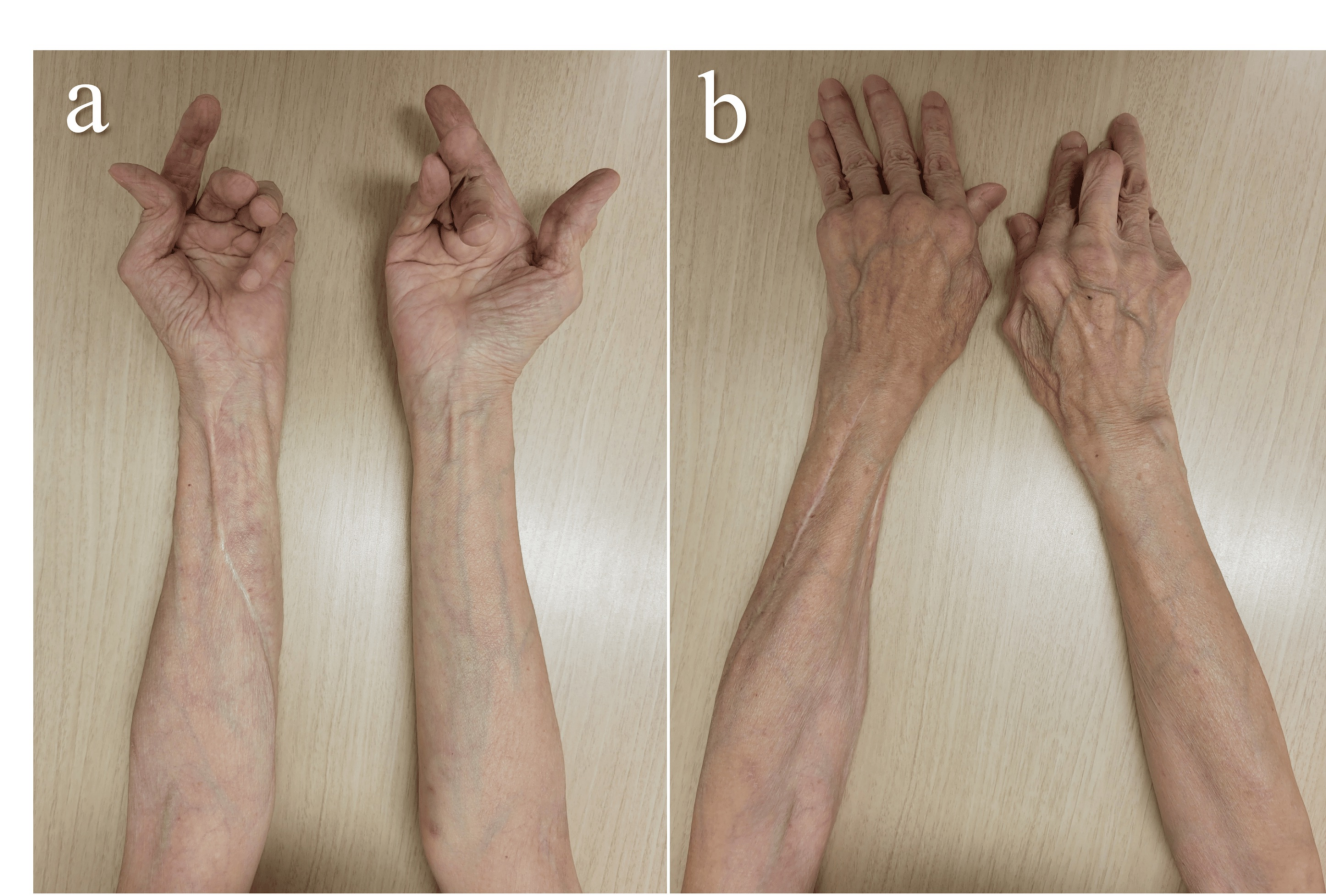
Appearance of the forearm at four years after surgery. (a) Volar view, (b) Dorsal view. There was no swelling, and wound healing was satisfactory. The fingers exhibited rheumatoid arthritis–related deformities.

The DLBCL achieved complete remission, and at the final follow-up four years after surgery, she remained disease-free. She reported no complications related to the forearm compartment syndrome and no apparent residual numbness. The Disabilities of the Arm, Shoulder and Hand score for disability and pain was 31 points, reflecting activity limitations due to rheumatoid arthritis. We recommended treatment for bilateral subcutaneous rupture of the extensor tendons of the third to fifth fingers and distal radioulnar joint disease; however, she declined further intervention due to advanced age (Figure 4).

## Discussion

We report the diagnostic and therapeutic course of a patient with primary skeletal muscle lymphoma presenting as forearm compartment syndrome. To date, only five cases of primary skeletal muscle lymphoma presenting as extremity compartment syndrome have been reported [3-5,7,8] (Table 2). Notably, all five reported cases first presented to orthopedic surgeons [3-5,7,8]. These cases manifested only local symptoms; therefore, diagnosis required two weeks to three months [3-5,7,8]. Primary muscle lymphoma has a poor prognosis because of its diagnostic difficulty and rapid progression [3-6]. All three reported cases of compartment syndrome caused by malignant lymphoma without postoperative chemotherapy resulted in death within 22 days after surgery [3-5]. This finding highlights the very limited window for diagnosis and initiation of treatment. Therefore, fasciotomy represents one of the very few opportunities to obtain diagnostic tissue, and it serves as a critical turning point for patient survival. Although double-expressor DLBCL is typically associated with poor prognosis [11], rapid diagnosis and appropriate initiation of therapy in our case led to complete remission and four years of relapse-free survival. To our knowledge, this represents the first report of long-term survival in double-expressor DLBCL presenting as compartment syndrome, underscoring its clinical significance.

**Table 2:** Summary of reported cases NHL, non-Hodgkin lymphoma; m-BACOD, modified regimen of bleomycin, doxorubicin (Adriamycin), cyclophosphamide, vincristine (Oncovin), and dexamethasone.

Authors	Age	Gender	Anatomic site	Histologic diagnosis	Initial presentation	Diagnostic trigger	Treatment	Follow-up
Lal et al. [3]	35	Woman	Right upper leg	NHL B cell type	Orthopedic surgery	Fasciotomy specimen	Supportive treatments	Died during hospitalization
Li et al. [4]	62	Woman	Left lower leg	B-cell lymphoma	Orthopedic surgery	Fasciotomy specimen	Supportive treatments	Died on day 22
Wang et al. [5]	68	Woman	Right thigh	Double-hit lymphoma	Orthopedic surgery	Fasciotomy specimen	Supportive treatments	Died on day 14
Chim et al. [7]	34	Man	Right forearm	Anaplastic Large Cell Lymphoma	Orthopedic surgery	Fasciotomy specimen	m-BACOD	4 years, disease-free survival
Southworth et al. [8]	80	Woman	Left leg	NHL	Orthopedic surgery	Fasciotomy specimen	Above-knee amptation	-

In non-traumatic compartment syndrome, the immediate priorities are the assessment of circulatory disturbance and infection, e.g., acute arterial occlusion, reperfusion injury, deep vein thrombosis, intramuscular bleeding due to coagulopathy, and necrotizing fasciitis [12]. Their differentiation should involve contrast-enhanced CT or MRI, in addition to laboratory testing. In our cases, however, there were no clear findings suggestive of circulatory disturbance or infection. Nevertheless, the patient’s history of COVID-19 vaccination, rheumatoid arthritis, and methotrexate use raised concern for viral myositis, dermatomyositis/polymyositis, and methotrexate-associated lymphoproliferative disorder [13]. All of these conditions can present with diffuse T1 isointensity and T2 hyperintensity in multiple muscles [14]; therefore, histopathological evaluation was necessary for a definitive diagnosis. Notably, these conditions generally respond to corticosteroid therapy [13,15]. Therefore, empirical corticosteroid administration may have advanced treatment and provided earlier symptom relief.

Although corticosteroids should preferably be initiated promptly after biopsy, their use before a confirmed diagnosis carries inherent risks. First, if the initial biopsy specimens had been inadequate, the diagnostic yield of subsequent examinations could have been reduced. Second, since corticosteroids can exacerbate infection, infectious conditions must be ruled out. Finally, systemic adverse effects of high-dose corticosteroids remain an important concern. Therefore, corticosteroid administration before histopathological confirmation should be carefully weighed against these risks, taking into account the severity of each individual case.

## Conclusions

This case illustrates a very rare primary skeletal muscle lymphoma presenting as compartment syndrome. Timely histopathological evaluation at the time of fasciotomy enabled early initiation of systemic chemotherapy, which contributed to a favorable outcome in this patient.

While this experience suggests that unexplained compartment syndrome should raise suspicion for underlying malignant disease, it remains uncertain how frequently such cases occur in clinical practice. Therefore, intraoperative muscle biopsy may be a prudent step when the clinical course is atypical. Further accumulation of cases will be required to clarify its diagnostic yield and establish standardized strategies.
